# Detection of invasive and native beetle species within trees by chemical analysis of frass

**DOI:** 10.1038/s41598-023-38835-x

**Published:** 2023-07-22

**Authors:** Nao Fujiwara-Tsujii, Hiroe Yasui

**Affiliations:** Division of Core Technology for Pest Control Research, Institute for Plant Protection, National Agriculture and Food Research Organization, Tsukuba, Ibaraki 305-8666 Japan

**Keywords:** Invasive species, Chemical ecology

## Abstract

In recent years, several invasive woodborers (Coleoptera: Cerambycidae) have been found in Japan. *Aromia bungii* is a worldwide important pest of fruits and ornamental species of the genus *Prunus*. It invaded Japan in the early 2010s and now causes heavy damage to stone fruit trees. *Anoplophora glabripennis* and *Apriona swainsoni* are destructive pests of street, ornamental and horticultural trees. The first step in intercepting these beetles is to detect their presence early in their infestation, as accurate identification is crucial for their management. Ejected frass is a major sign of infestation and likely holds information on the insect. We focused on chemicals in both larvae and frass, and conducted a GC–MS analysis of these three invasive beetles and the native *Anoplophora malasiaca*. In all four species, 4 or 5 species-specific hydrocarbons were detected in both larvae and frass. These results indicate that analysis of hydrocarbons in frass could allow definitive detection of invasive wood-boring pests.

## Introduction

Invasive beetle species, especially in the family Cerambycidae, are important pests worldwide^1^. Their larvae bore deeply into trees, shrubs and wood products. Invasive species can easily find new hosts and establish new habitats in invaded areas. In recent years, three invasive longhorn beetles species have been found in Japan, but there is not yet enough ecological information on them, such as life cycle and host range. And no effective control methods have also been established yet. The first important step in their eradication is to detect their presence within trees early in their infestation. Then, their accurate identification is crucial for their management, such as the selection of suitable pesticides, to prevent more damage.

*Aromia bungii* (Faldermann) (Coleoptera: Cerambycidae), the red-necked longhorn beetle, was first recognized in Japan in the early 2010s^2^, and is now (March 2023) present in 13 of the 47 prefectures^3–5^. It is an important wood-boring pest of fruits and ornamental species of the genus *Prunus*, including the culturally significant cherry blossom, which attracts tourists from both Japan and overseas. Horticultural species, especially peaches, are also heavily damaged by this species^3^.

*Anoplophora glabripennis* (Motschulsky) (Coleoptera: Cerambycidae), the Asian long-horned beetle, a devastating pest, was confirmed in Japan in 2020 and 2021^6–12^ and is already present in North America and Europe^13,14^. It was included in a list of the 100 of the World’s Worst Invasive Alien Species^15^. It poses a serious threat to landscape trees, such as maple (*Acer*), poplar (*Populus*), and willow (*Salix*), often killing host trees by vigorous larval feeding^16^. In invaded regions, aggressive containment programs have been carried out, such as removal and destruction of all trees with signs of infestation^16,17^. Japan is home to a native *Anoplophora* beetle, *A*. *malasiaca* (Thomson), the white-spotted longicorn beetle^18^. It is synonymized with *Anoplophora chinensis*^19^, although its taxonomy is confusing. Here, we use ‘*A*. *malasiaca*’ refer to the Japanese population. It is a serious pest of horticultural tree, such as citrus (*Citrus*), apple (*Malus*) and pear (*Pyrus*), and of street trees, such as oriental planetree (*Platanus*) and willow^18,20^. It has a very wide range of host plants, 108 species in 73 genera^21^. The invasive and native *Anoplophora* beetles look similar and share some host plants (e.g. *Salix*, *Cercidiphyllum*), most people could not distinguish them. It can be tricky to distinguish them for control, so the early detection method is eagerly awaited.

*Apriona swainsoni* (Hope) (Coleoptera: Cerambycidae) was confirmed in Fukushima prefecture in 2021^22^. It is a serious pest of *Styphnolobium japonicum* in China^23^. In Japan, it frequently attacks street trees including *S*. *japonicum*, but especially does *Maackia amurensis*.

Species identification of beetle larvae is difficult because of their morphological similarities. For determination, larvae are often taken out from the tree and reared in the laboratory until adulthood, which can take several months^24^. Moreover, the collection of larvae from trees can injure the host tree as well as the larvae^25^. On the other hand, frass ejection from trees infested by pest insects is often observed in the field. Frass includes woody materials and sometimes faeces of infesting insects.

Invasive beetles can be identified from their genetic information. Analysis of frass by real-time PCR confirmed the presence of *A*. *bungii*^25^ and another wood-boring pest^26^. Although this method guarantees accuracy of species identification, the stability of genetic information in the field is not assured under the tough conditions of rain and sunlight. To develop better methods for identifying infesting beetles under tough field conditions, we focused on the hydrocarbons in their frass. Among possible chemical components in frass, hydrocarbons are synthesized by insects and are chemically more stable than polar compounds and DNA. Insect cuticular hydrocarbons (CHCs) have roles in maintaining the insect’s water balance and acting as signalling molecules for mate recognition and chemical communication^27–29^. CHCs of many adult insects are reported to be species specific^30^. In contrast, only a few reports of larval CHCs were found: one on blowfly larvae^31^, one on *Tribolium confusum* (Coleoptera, Tenebrionidae)^32^, and some on parasitic lepidopteran larvae which penetrate ant nests^33^. These reports describe age-dependent changes in larval CHCs (blowfly), kairomonal activity (*T*. *confusum*), or chemical camouflage.

Here, we compared profiles of CHCs of larvae and frass of three invasive and one native beetles and confirmed these commonalities. Based on these comparisons, we could reveal that analysis of hydrocarbons in frass could contribute to detecting beetle species in trees. Furthermore, we demonstrate the validity of our method by using frass samples of *A*. *bungii*. We discuss the possible use of hydrocarbon analysis for the identification of infesting species.

## Results

### Common hydrocarbons detected in larvae and frass of four beetle species

Table 1 lists hydrocarbons commonly detected in frass and larvae of each species, with the Kovát’s Index and molecular or fragment ions used for their identification. Double-bond positions of unsaturated hydrocarbons were determined only in *A*. *bungii*. Some hydrocarbons were detected in multiple species; for example, *n*-tricosane was detected in *A*. *bungii*, *A*. *glabripennis* and *A*. *swainsoni*. All four species have species specific hydrocarbons: for example, 6,9-C_25:2_ and 6,9-C_27:2_ in *A*. *bungii*; C_27:1_ in *A*. *glabripennis*; C_28_, 4Me-C_28_ and C_29_ in *A*. *malasiaca*; and C_23:1_ and C_25:1_ in *A*. *swainsoni*.

**Table 1:** Hydrocarbons both detected in larvae and frass of each beetle species.

Commonly detected hydrocarbons	Abbreviation	KI (HP-5ms column)	Characteristic ion (molecule/fragment)
*Aromia bungii*			
* n*-Tricosane	C_23_	2300	324
* n*-Tetracosane	C_24_	2400	338
6,9-Pentacosadiene	6,9-C_25:2_	2474	348
* n*-Pentacosane	C_25_	2500	352
6,9-Heptacosadiene	6,9-C_27:2_	2676	376
*Anoplophora glabripennis*			
* n*-Tricosane	C_23_	2300	324
* n*-Tetracosane	C_24_	2400	338
* n*-Pentacosane	C_25_	2500	352
Heptacosene	C_27:1_	2674	378
* n*-Heptacosane	C_27_	2700	380
*Anoplophora malasiaca*			
* n*-Heptacosane	C_27_	2700	380
* n*-Octacosane	C_28_	2800	394
4-Methyloctacosane	4Me-C_28_	2868	365, 393
* n*-Nonacosane	C_29_	2900	408
*Apriona swainsoni*			
Tricosene	C_23:1_	2276	322
* n*-Tricosane	C_23_	2300	324
Pentacosene	C_25:1_	2476	350
* n*-Pentacosane	C_25_	2500	352
* n*-Heptacosane	C_27_	2700	380

### Aromia bungii

**Figure 1:**
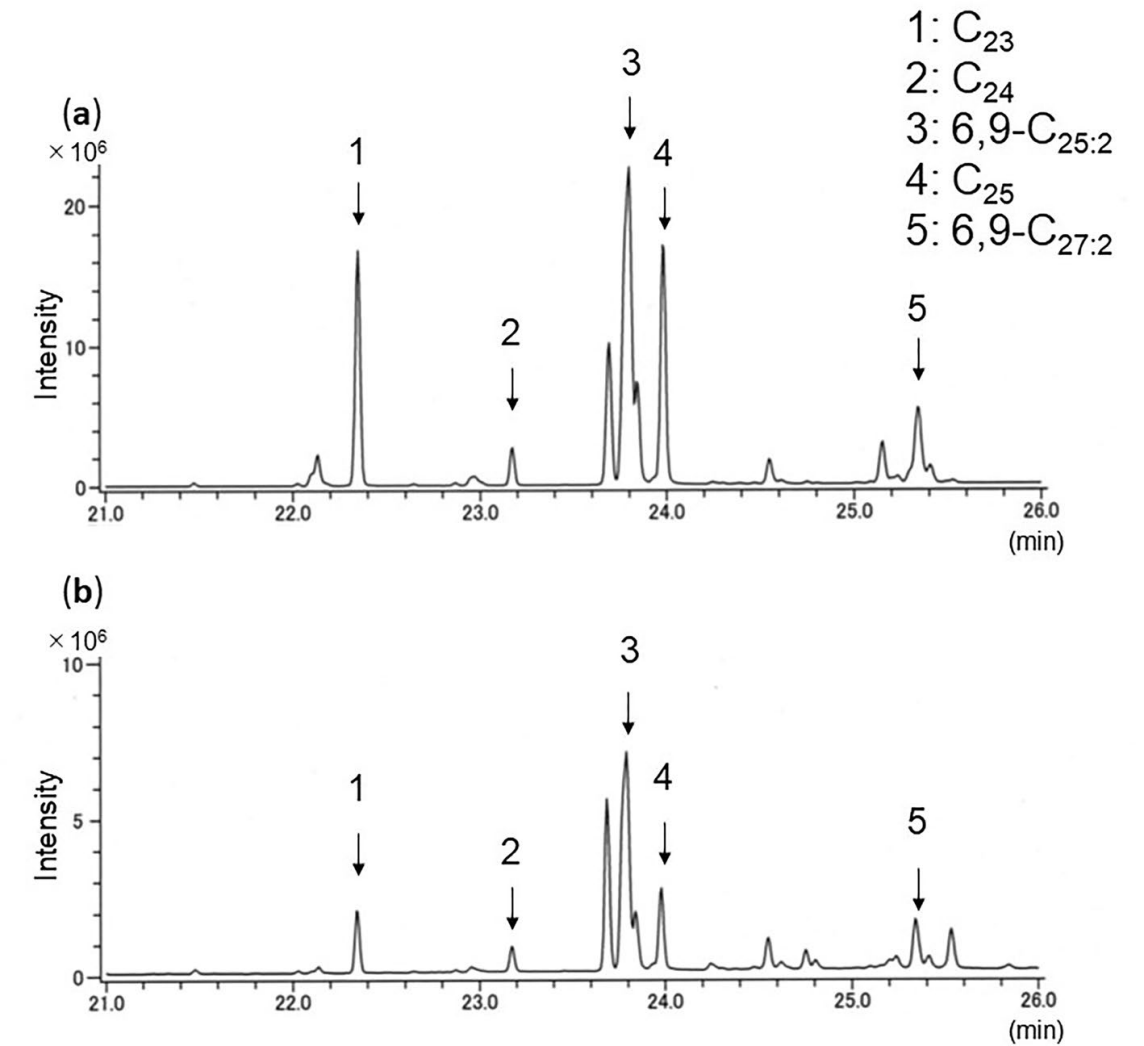
Total ion chromatogram of GC/MS profiles of hydrocarbons of (**a**) larvae and (**b**) frass of *A*. *bungii* from plum tree. C_23_, *n*-tricosane; C_24_, *n*-tetracosane; 6,9-C_25,2_, 6,9-pentacosadiene; C_25_, *n*-pentacosane; 6,9-C_27,2_; 6,9-heptacosadiene.

Both *A*. *bungii* larvae and frass showed the same five hydrocarbons (Table 1; Fig. 1, numbered peaks): three were saturated (C_23_, C_24,_ C_25_) and two were unsaturated (diene: 6,9-C_25:2_, 6,9-C_27:2_). C_23_ and C_25:2_ were always detected at strong intensity.

### Anoplophora glabripennis

**Figure 2:**
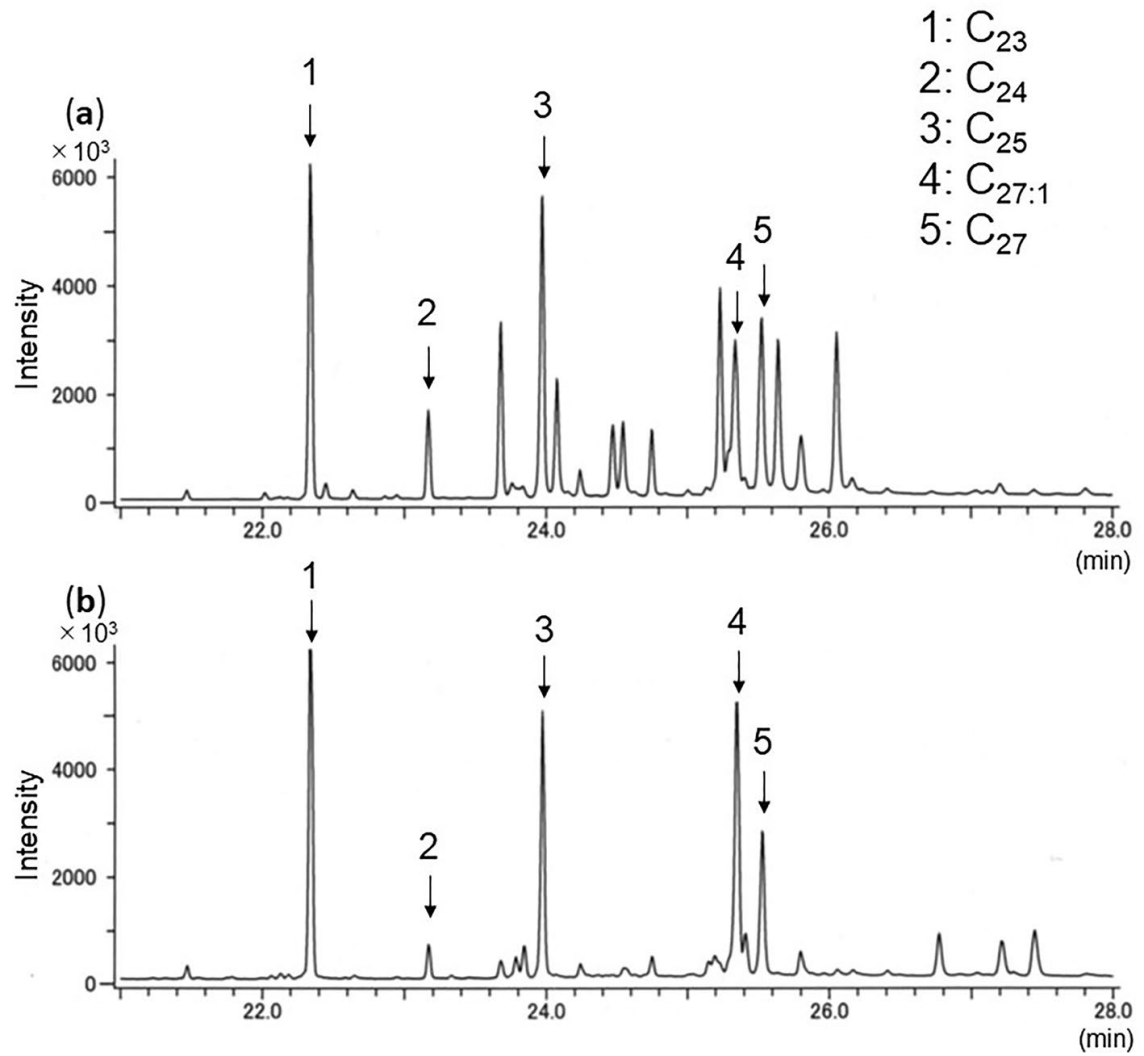
Total ion chromatogram of GC/MS profiles of hydrocarbons of (**a**) larvae and (**b**) frass of *A*. *glabripennis* from willow tree. C_23_, *n*-tricosane; C_24_, *n*-tetracosane; C_25_, *n*-pentacosane; C_27,1_, heptacosene; C_27_, *n*-heptacosane.

Both *A*. *glabripennis* larvae and frass showed the same five hydrocarbons (Table 1; Fig. 2, numbered peaks): four were saturated (C_23_, C_24,_ C_25_, C_27_) and one was unsaturated (monoene: C_27:1_). C_23_ and C_25_ were always detected at strong intensity.

### Anoplophora malasiaca

**Figure 3:**
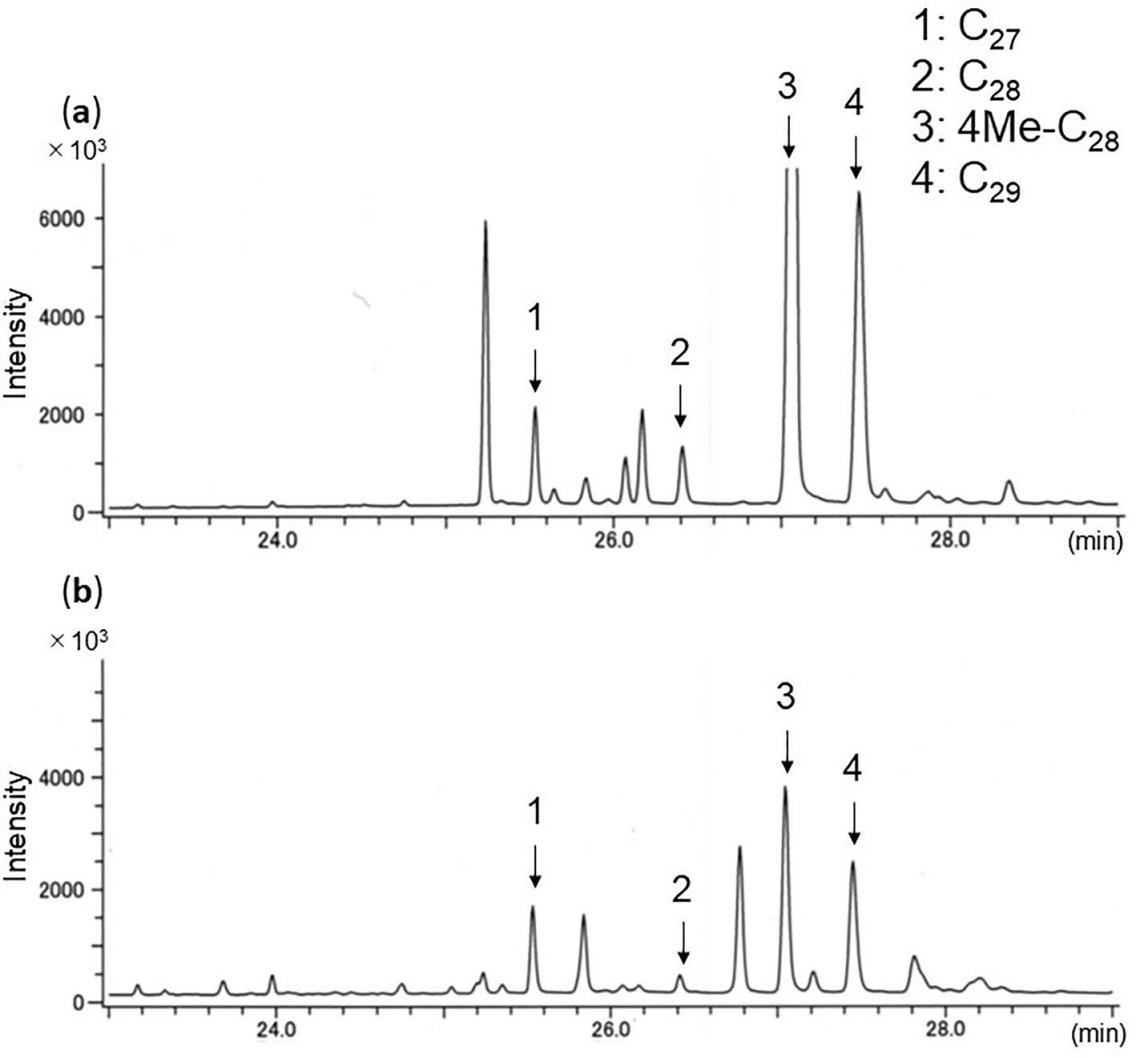
Total ion chromatogram of GC/MS profiles of hydrocarbons of (**a**) larvae and (**b**) frass of *A*. *malasiaca* from willow tree. C_27_, *n*-heptacosane; C_28_, *n*-octacosane; 4Me-C_28_, 4-methyloctacosane; C_29_, *n*-nonacosane.

Both *A*. *malasiaca* larvae and frass showed the same four hydrocarbons (Table 1; Fig. 3, numbered peaks): three were saturated (C_27_, C_28,_ C_29_) and one was a monomethyl (4Me-C_28_). 4Me-C_28_ and C_29_ were always detected at strong intensity.

### Apriona swainsoni

**Figure 4:**
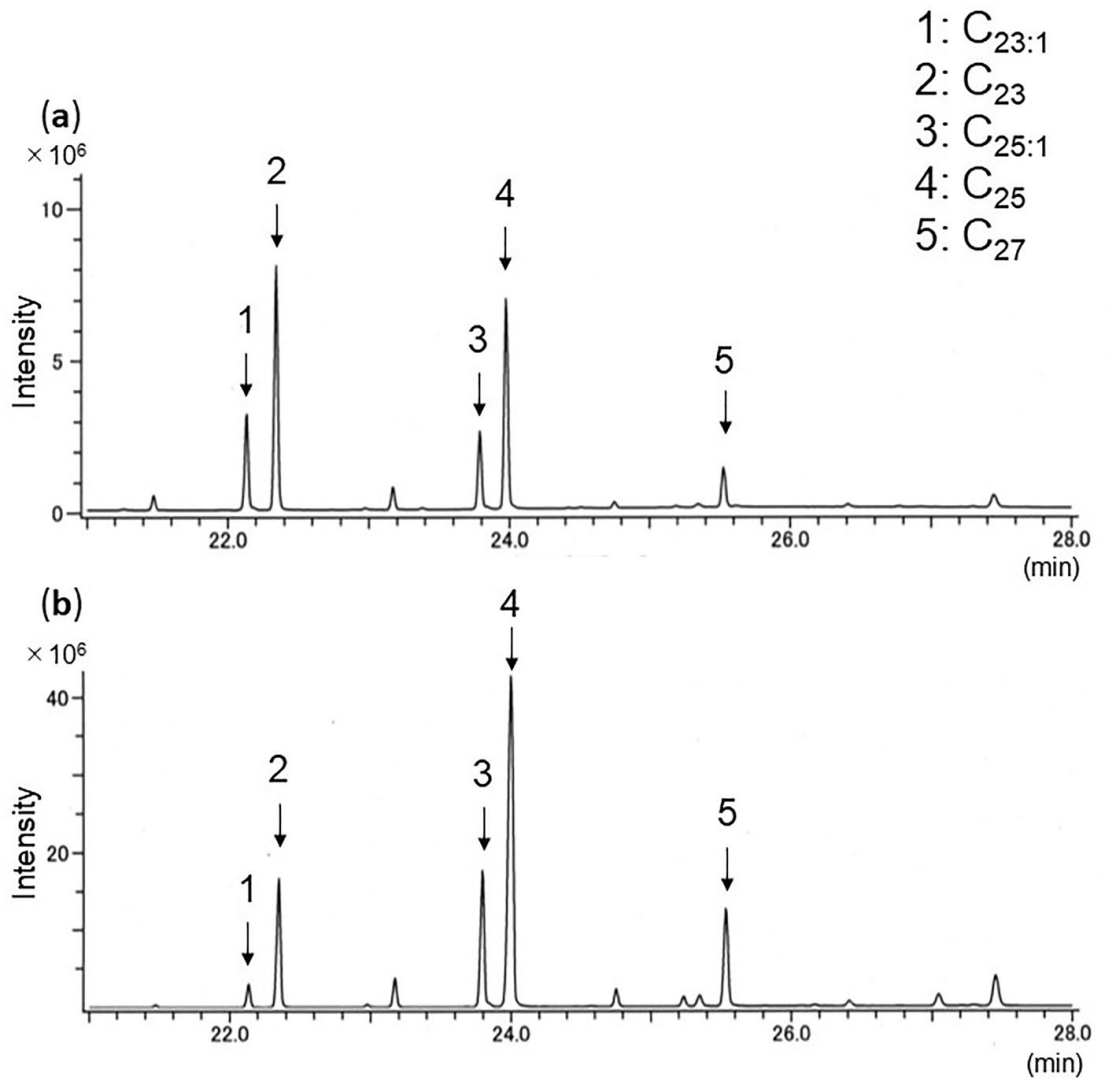
Total ion chromatogram of GC/MS profiles of hydrocarbons of (**a**) larvae and (**b**) frass of *A*. *swainsoni* from *M*. *amurensis*. C_23,1_, tricosene; C_23_, *n*-tricosane; C_25,1_, pentacosene; C_25_, *n*-pentacosane; C_27_, *n*-heptacosane.

Both *A*. *swainsoni* larvae and frass showed the same five hydrocarbons (Table 1; Fig. 4, numbered peaks): three were saturated (C_23_, C_25_, C_27_) and two were unsaturated (monoene: C_23:1_, C_25:1_). C_25_ and C_25:1_ were always detected at strong intensity.

### Comparison of detected hydrocarbons among 4 different beetle species

Every beetle’s larva and frass had some hydrocarbons (Table 1). Table 2 summarizes the hydrocarbons detected in each species.

**Table 2:** Comparison of detected hydrocarbons among 4 different beetle species.

Detected hydrocarbons		*Aromia bungii*	*Anoplophora glabripennis*	*Anoplophora malasiaca*	*Apriona swainsoni*
Tricosene	C_23:1_				+
*n-*Tricosane	C_23_	++	++		+
*n*-Tetracosane	C_24_	+	+		
Pentacosene	C_25:1_				++
6,9-Pentacosadiene	6,9-C_25:2_	++			
*n*-Pentacosane	C_25_	+	++		++
Heptacosene	C_27:1_		+		
6,9-Heptacosadiene	6,9-C_27:2_	+			
*n*-Heptacosane	C_27_		+	+	+
*n*-Octacosane	C_28_			+	
4-Methyloctacosane	4Me-C_28_			++	
*n*-Nonacosane	C_29_			++	

++: Peaks always detected at large intensity (occupied > 15% of numbered peaks).

### Hydrocarbon analysis of field-collected frass of *Aromia bungii* from two tree species

**Figure 5:**
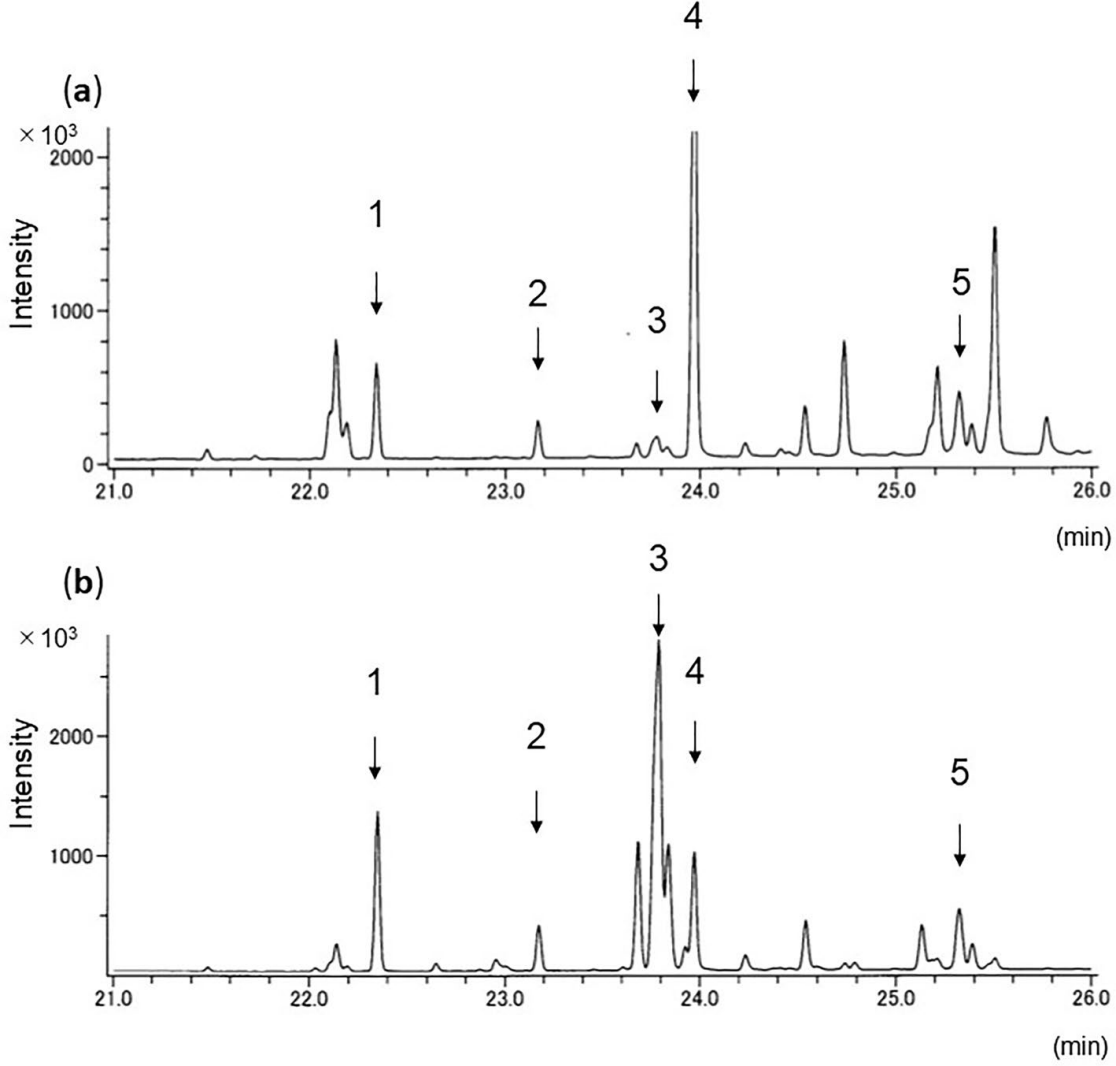
Total ion chromatogram of GC/MS profiles of hydrocarbons of *A*. *bungii* frass collected from (**a**) cherry tree in the field and (**b**) peach tree in the laboratory. Peak numbers indicate the same hydrocarbon components shown in Fig. 1.

The field-collected frass of *A*. *bungii* larvae (over 2-month-old larvae) showed the same profile as that under laboratory conditions (Figs. 1a, 5a,b). The same five hydrocarbons were detected from the frass of both tree species (Fig. 5a, cherry tree; b, peach tree, numbered peaks).

### Hydrocarbon analysis of sawdust and frass from plum tree infested by *A*. *bungii*

**Figure 6:**
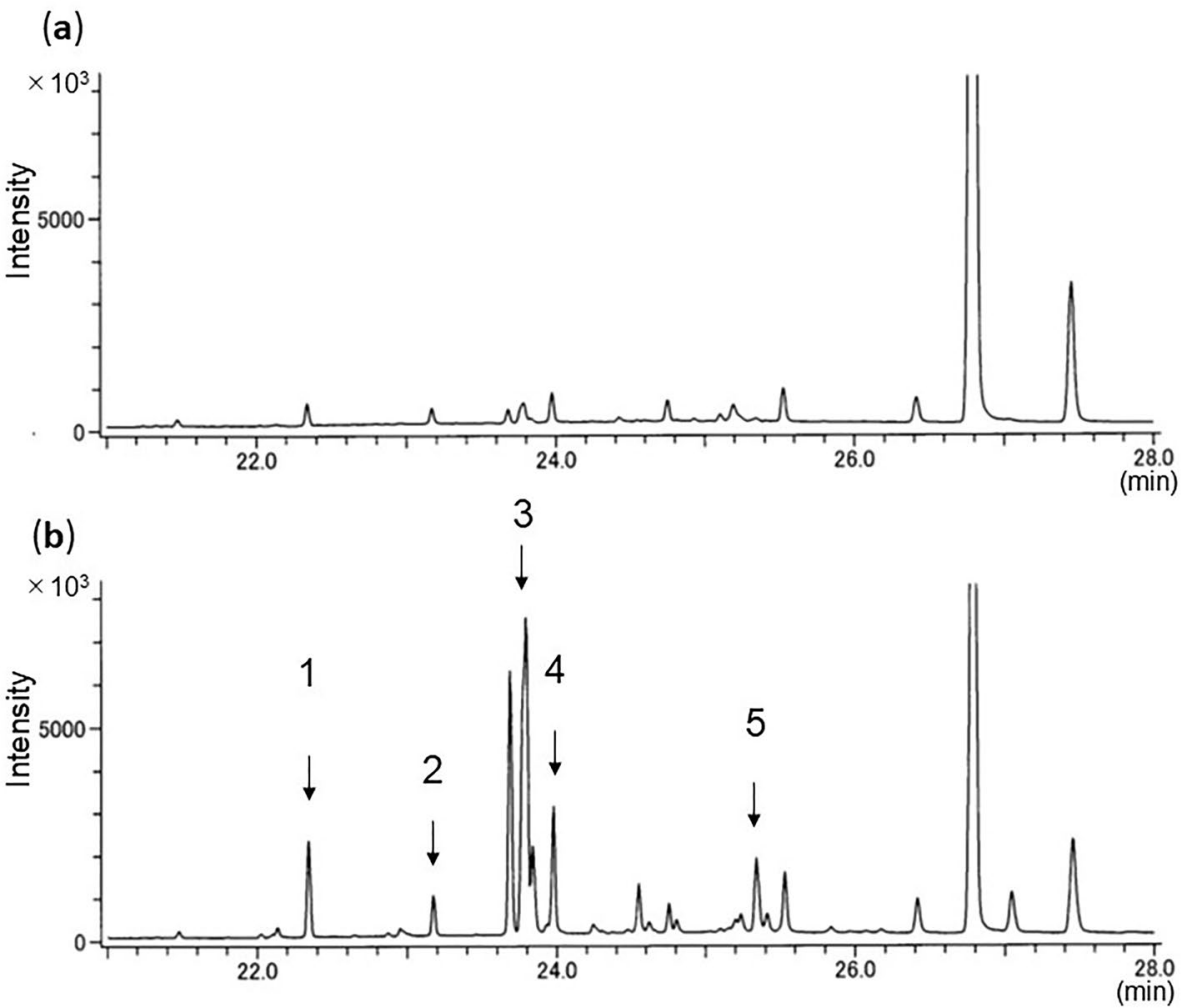
Total ion chromatogram of GC/MS profiles of hydrocarbons of (**a**) sawdust and (**b**) frass made by* A*. *bungii*. All samples were collected from plum trees. Peak numbers indicate the same hydrocarbon components shown in Fig. 1.

Larval-specific hydrocarbons were not detected in the sawdust extract (Fig. 6a). Only frass showed the five characteristic hydrocarbons (Fig. 6b, arrows).

### Time-course hydrocarbon analysis of *Aromia bungii* frass

**Figure 7:**
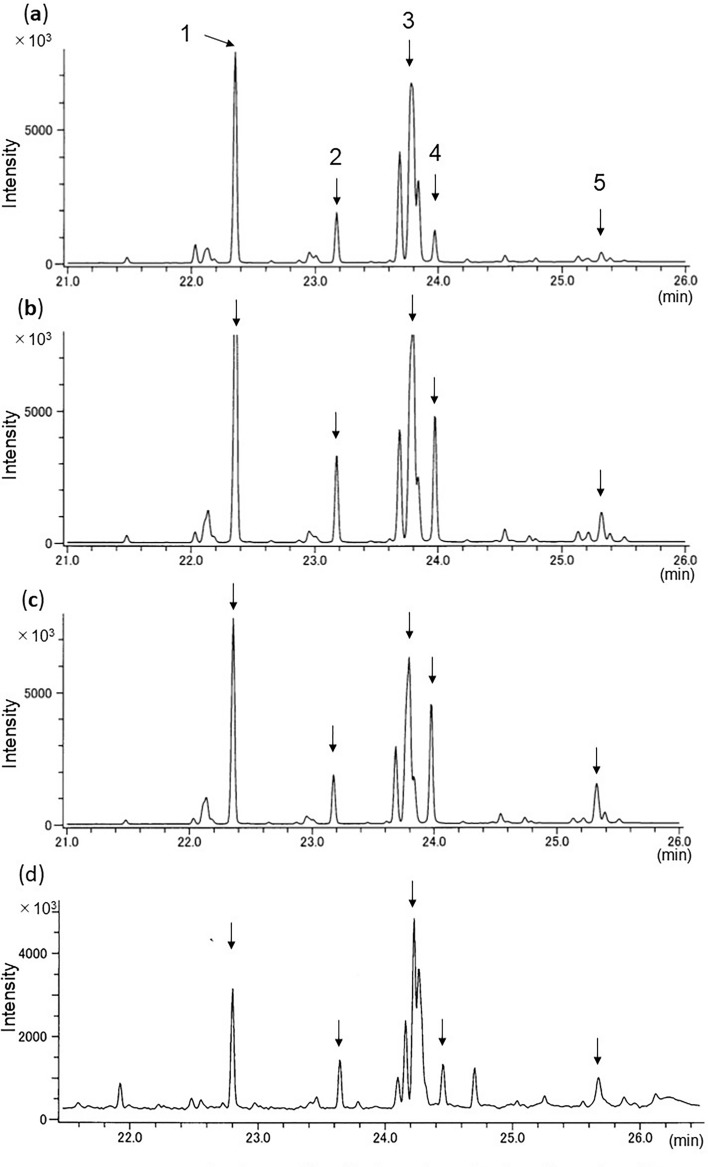
Total ion chromatograms of GC/MS profiles of hydrocarbons of *A*. *bungii* frass ejected by larvae of different age. Each chromatogram shows the frass hydrocarbon profiles of larvae which after hatching (**a**) week 1; (**b**) week 3; (**c**) week 5; (**d**) around week 36 (supposed to overwintered, 9-month-old larvae). Peak numbers indicate the same hydrocarbon components shown in Fig. 1.

Weekly analysis of *A. bungii* frass showed that the hydrocarbon profiles were unchanged over 5 weeks after larvae hatching (Fig. 7a, week 1; b, week 3; c, week 5). As a reference data, field-collected frass ejected by over-wintered, 9-month larvae included same species-specific hydrocarbons (Fig. 7d). *Aromia bungii*-specific hydrocarbon profiles were detected through the larval period.

## Discussion

We developed a new method to detect three invasive and one native beetle species from frass ejected from infested trees. Extraction of hydrocarbons and GC–MS analysis correctly identified the beetle species in the trees. Every sample showed clear total ion chromatograms, and all detected peaks could be chemically characterized. Four or five hydrocarbons common to both larvae and frass were consistently detected within species. As reported of other insect species^28^, CHC profiles of tested larvae differed among species. We also got the same hydrocarbon profiles of *A*. *bungii* frass from laboratory (Fig. 1) and field samples (Fig. 5). This result reveals that our method could be applied to both laboratory and field samples.

CHCs of insects consist of a complex mixture of straight-chain (saturated), unsaturated, and methyl-branched components with over 20 carbon atoms^28^. In *A*. *bungii*, *A. malasiaca* and *A*. *glabripennis*, chemical profiles of CHCs of adults and their larvae are not common [^34^ (cf. our data), ^35,36^]. There is no information on the roles of larval CHCs of these beetles in chemical communication, but some may have kairomonal activity against parasitoids. *Dastarcus helophoroides* (Coleoptera: Bothrideridae) is a larval predator of cerambycid forest tree pests, and its mass release is one option for controlling beetles^37^. Volatile chemicals from the host tree or insects play a key role in host searching from a distance^37^, although, commonly detected CHCs in this study are slightly volatile and almost non-volatile, there is a possibility to have close-distant or contact kairomonal activity.

Currently, the most accurate method to identify infesting beetle species is to collect larvae and rear them to adulthood, because beetle larvae look very similar^38^. However, this method damages trees. Instead, if ejected frass is present, hydrocarbon analysis could be used to identify them without further damage to the tree. We confirmed the validity of our method with *A*. *bungii* because of sample availability. The results of the chemical analysis of *A*. *bungii* frass from plum and peach (Figs. 1b, 5b), plum (Fig. 6b), and cherry trees (Fig. 5a) show that our method can work with different tree species. Even within their ‘first week of feeding or just after hatch, frass was seen. Only 1 mg equivalent of frass extract injected into the GC/MS could determine the profiles of its typical hydrocarbons. Thus, our method could be applied very early in larval infestation; the earlier an infestation can be detected, the earlier it can be treated, avoiding further damage. This is crucial to reducing the damage by wood-boring beetles to economically, culturally or historically important trees. Throughout our experiments, larvae-frass common hydrocarbons were stably detected from early stage (1–5 weeks, Fig. 7a–c), 2-month over (Fig. 5a,b) and 9-month over (Fig. 7d). As is known in other insects, CHCs can change over the larval stage^31^. If the same tendency existed in the beetles, our method could allow us to estimate larval age or size. The entire larval stage changes of their CHCs composition should be investigated in our future work.

We could detect hydrocarbons in almost all *A*. *bungii* and *A*. *swainsoni* frass samples. In the case of *A*. *glabripennis* and *A*. *malasiaca*, however, the frass sometimes did not include detectable amounts of hydrocarbons. In our interpretation, the difference may be caused by larval behaviour. *Aromia bungii* and species of *Apriona* (including *A*. *swainsoni*) continually make excretory holes for ejecting their frass^39,40^. During our preliminary experiment, *Aromia* larvae used their body segments to eject the frass. This active ejection of frass may transfer their CHCs to the frass. On the other hand, this behaviour has not been reported in *Anoplophora* larvae and we haven’t observed it. We could detect *A*. *malasiaca*-specific hydrocarbons in frass from willow twigs (Fig. 3), but not in other trials using fresh or live trees. One possibility is that when feeding on spatially restricted dry material (such as cut twigs), *A*. *malasiaca* larvae have to maintain their living and feeding space by ejecting frass. As a result of that behaviour, their CHCs might often be undetectable.

Many other sympatric insects eject frass in the same way as our beetles. To improve risk management of trees worldwide, we should accumulate examples of insect cuticular and frass hydrocarbon profiles not only of Coleopteran but also of other frass-ejecting insect species.

## Materials and methods

### Collection and rearing of adult beetles

#### *Aromia bungii*

Adult *A*. *bungii* were collected by hand from peach groves in Sano city, Tochigi Prefecture, Japan, in late June 2022.

#### *Anoplophora glabripennis *and* A. malasiaca*

Adult *A*. *glabripennis* and *A. malasiaca* were collected by hand from street trees such as *Salix* spp. (willow) and *Cercidiphyllum japonicum* in Tsukuba, Omitama and Ishioka cities, Ibaraki Prefecture, in June and July 2022.

#### *Apriona swainsoni*

Adult *A*. *swainsoni* were collected by hand from *Maackia amurensis* in Tenei village and Koriyama city, Fukushima Prefecture, in August 2022. Some emerged from host plants (*M*. *amurensis*) cut back in Koriyama city in May 2022.

*Aromia bungii*, *A. glabripennis* and *A*. *malasiaca* beetles were individually reared in clear plastic cups (ca. 11 cm diam. × 9.5 cm height) at 24 °C under a 15L:9D photoperiod, illuminated by fluorescent lamps. All females mated at least once in our laboratory. *A. bungii* were fed artificial diet for rhinoceros beetle (Fujikon Co. Ltd, Osaka, Japan); and *A*. *glabripennis* and *A. malasiaca* were fed willow and citrus twigs, renewed every 5 days. *Apriona swainsoni* were reared in meshed cages (22 cm × 42 cm × 42 cm), together with two or three branches (30 cm long, 10 cm diameter) of *M*. *amurensis* for feeding and oviposition, renewed every 5 days.

### Frass collection

#### *Aromia bungii*

In the laboratory, small branches of plum or peach (5 cm diam., 10 cm long) were partially wrapped in 1-cm-wide Parafilm (Bemis Flexible Packaging, Chicago, IL, USA), and females of *A*. *bungii* were housed on them for 2 days. Eggs laid between the bark and the Parafilm were collected and laid on wet filter paper (9 cm diam., Toyo Roshi Kaisha, Ltd, Tokyo, Japan). Eggs on filter paper were placed together in a plastic Petri dish (9 cm diam. × 2 cm height) at 24 °C under a 15L:9D photoperiod, illuminated by fluorescent lamps for 10 days. Newly hatched larvae were placed on new twigs of plum. After a few weeks, larvae began to eject frass, which was then collected by forceps into glass vials weekly for 5 weeks. Frass was also collected from peach groves and cherry trees in Sano and Ashikaga city on October 4th, 2022, Tochigi prefecture, where we collected the *A*. *bungii*. These field collected frass were expected to be ejected by over 2-month larvae of *A*. *bungii*. Frass of over-wintered larvae also collected on May 25th, 2022 from woods bring back from peach groves in Sano city in December 2021 and maintained in the laboratory (9-month over larvae).

#### *Anoplophora glabripennis *and* A. malasiaca*

Females of each species were individually kept in clear plastic cups (ca. 11 cm diam. × 9.5 cm height) together with host twigs, mainly willow (3 cm diam., 5 cm long). Eggs laid under the bark were collected and laid on wet filter paper. Eggs on filter paper were placed together in a plastic Petri dish as above for 10 days. Newly hatched larvae were placed on new plum or willow twigs. Ejected frass was collected as above in our laboratory (*A. glabripennis* and *A. malasiaca*, three samples) and from two frass samples were collected from *Cercidiphyllum japonicum* in Tsukuba from the field (*A. glabripennis*).

#### *Apriona swainsoni*

Males and females of *A*. *swainsoni* were kept together in small mesh cages (28 cm × 30 cm × 18 cm). Females laid eggs into *Maackia Amurensis* branches. The branches were maintained as above. After a few weeks, larvae began to eject frass, which was collected as above in our laboratory (two samples) in 2022. In 2023, three larvae and frass were collected from the infested wood cut in Fukushima prefecture.

### Extraction and purification of hydrocarbons from frass and larvae

All frass was freeze-dried for 16 h at − 30 °C before extraction. Dried samples were weighed and then extracted in 5–8 mL of *n*-hexane (HPLC grade, Fujifilm-Wako, Osaka, Japan) for 5 min. Sample sizes of each frass were 10 for *A*. *bungii*, 5 for *A*. *glabripennis* and *A*. *swainsoni*, 3 for *A. malasiaca*.

Larvae were placed on filter paper for 20 min to void faeces and then frozen (− 30 °C). Each larva was put into a glass vial (5–12 mL) and extracted with *n*-hexane for 5 min. Sample sizes of each larva were 5 for *A*. *bungii*, 5 for *A*. *glabripennis*, 3 for *A. malasiaca*, and 5 for *A*. *swainsoni*.

Hexane extracts were filtered and poured through a silica gel column (Wakogel C-300, 0.5 g, Fujifilm-Wako). The final volume of extracts after concentration depended on frass volume or larval size, but 0.001 g equivalent of frass sample or 0.01 larva equivalent dissolved in 1 µL *n*-hexane was injected into the GC–MS.

### Comparison of hydrocarbons in frass and larvae on plum tree in the laboratory

Females of *A*. *bungii* were introduced into a meshed cage with a plum tree in a pot (2 years old, 100 cm high) and allowed to lay freely for 2 days. After 2–3 weeks, hatched larvae started to eject frass. At the same time, sawdust was collected by hand-sawing for analysis to confirm the components of the host plant. Frass and sawdust were extracted as above.

### Gas chromatography/mass spectrometry (GC/MS) analyses

GC/MS analyses were performed on an Agilent 7890N GC system (Agilent, Santa Clara, CA, USA) interfaced to a JEOL JMS-T100GC time-of-flight mass spectrometer (JEOL, Tokyo, Japan) in EI mode at 70 eV at 220 °C. Samples were injected in splitless mode at 220 °C for 1 min, with helium as the carrier gas in constant flow mode (1.1 mL/min). The capillary column, a DB-5MS (30 m × 0.25 mm ID × 0.25 μm film thickness; Agilent), was linked to an MS, and the GC oven temperature was held at 40 °C for 1 min, increased from 40 to 250 °C at 10 °C/min and then held at the final temperature for 10 min.

### Identification of hydrocarbons in larvae and frass

Hydrocarbons were identified by the mass spectrum and the Kovát index of each peak^41^. A reference hydrocarbon mixture that included saturated, even-numbered hydrocarbons from C_12_ to C_32_ was analysed as above, with reference to KIs of standard compounds. Only *A*. *bungii* yielded diene components in sufficient amount for derivatization to decide the position of two double bonds. Diene components were partially reduced with hydrazine and epoxidated by *m*-chloroperoxybenzoic acid. The resultant epoxide mixture was analysed by GC–MS, and the position of the double bonds was determined^42^.

## Supplementary Information

Supplementary Information 1.

Supplementary Information 2.

Supplementary Information 3.

Supplementary Information 4.

Supplementary Information 5.

Supplementary Information 6.

Supplementary Information 7.

Supplementary Information 8.

## Data Availability

The data presented in this study are available on request from the corresponding author.
