# Supplementary figures and images for: Detection of invasive and native beetle species within trees by chemical analysis of frass
Source: Sci Rep. 2023 Jul 22;13:11837. doi: 10.1038/s41598-023-38835-x (PMC10363158; doi:10.1038/s41598-023-38835-x)

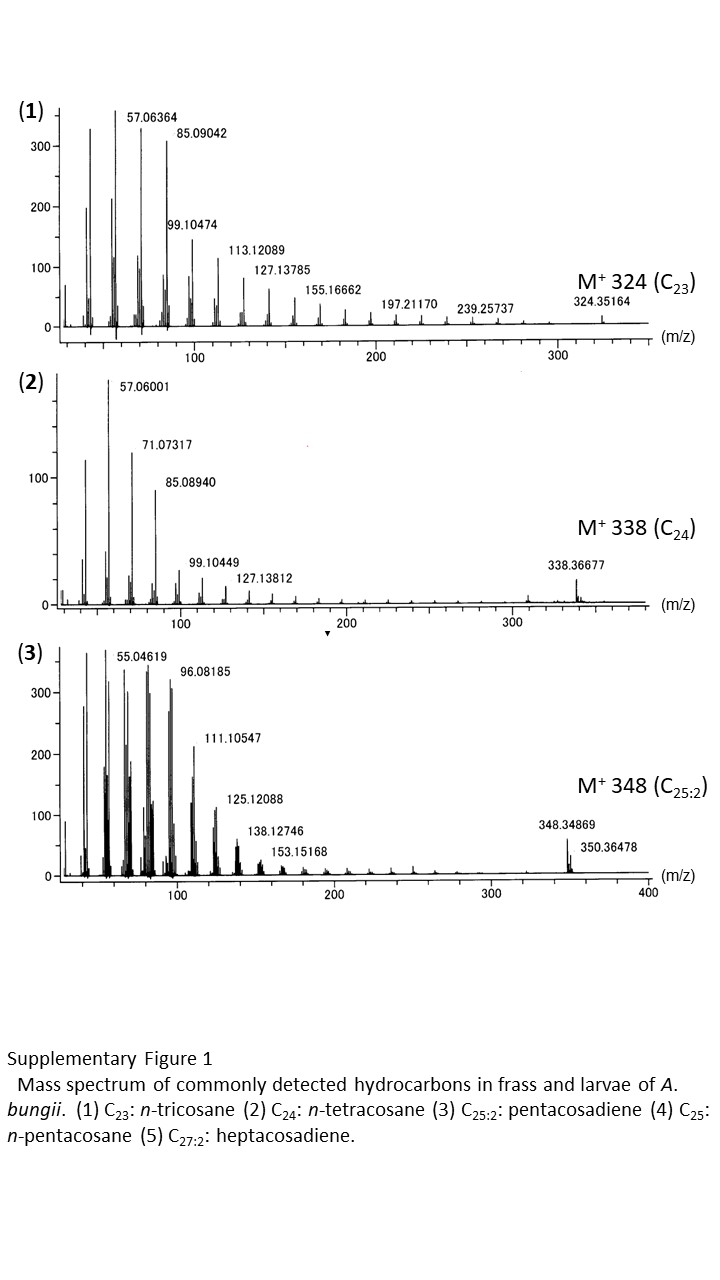

Supplement: Supplementary file 1 — Supplementary Information 1. [file 41598_2023_38835_MOESM1_ESM.jpg]

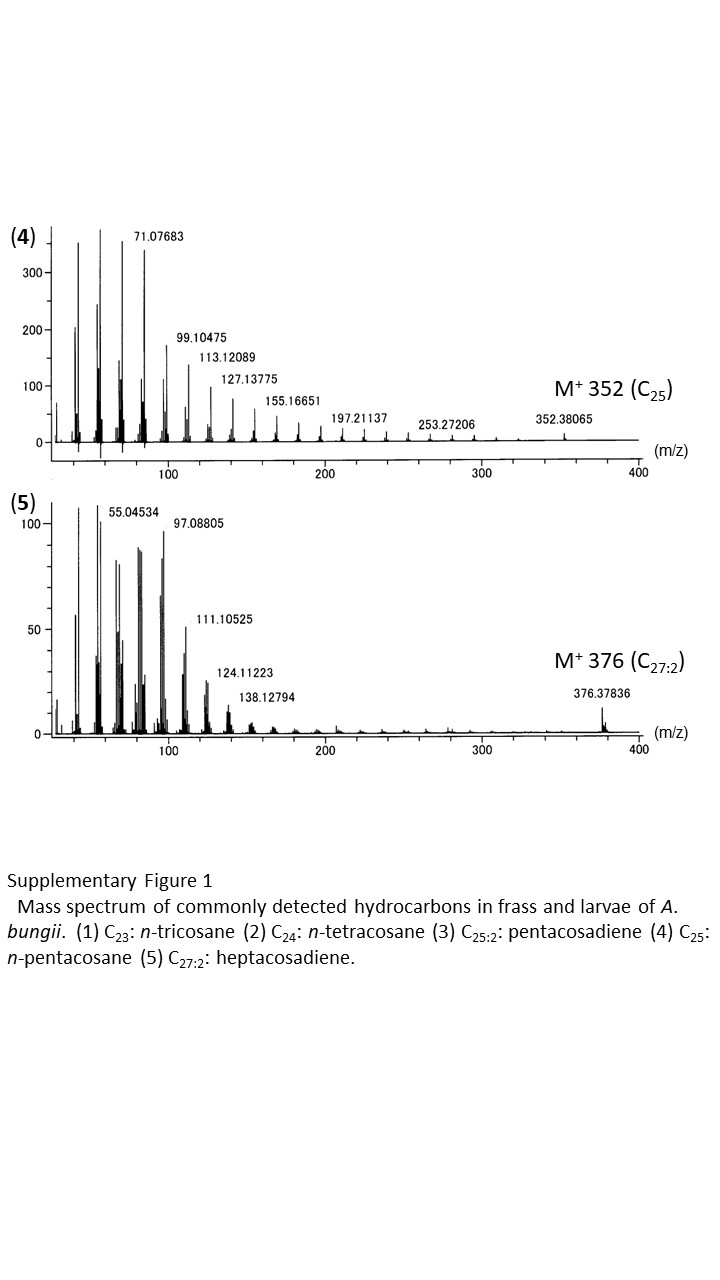

Supplement: Supplementary file 2 — Supplementary Information 2. [file 41598_2023_38835_MOESM2_ESM.jpg]

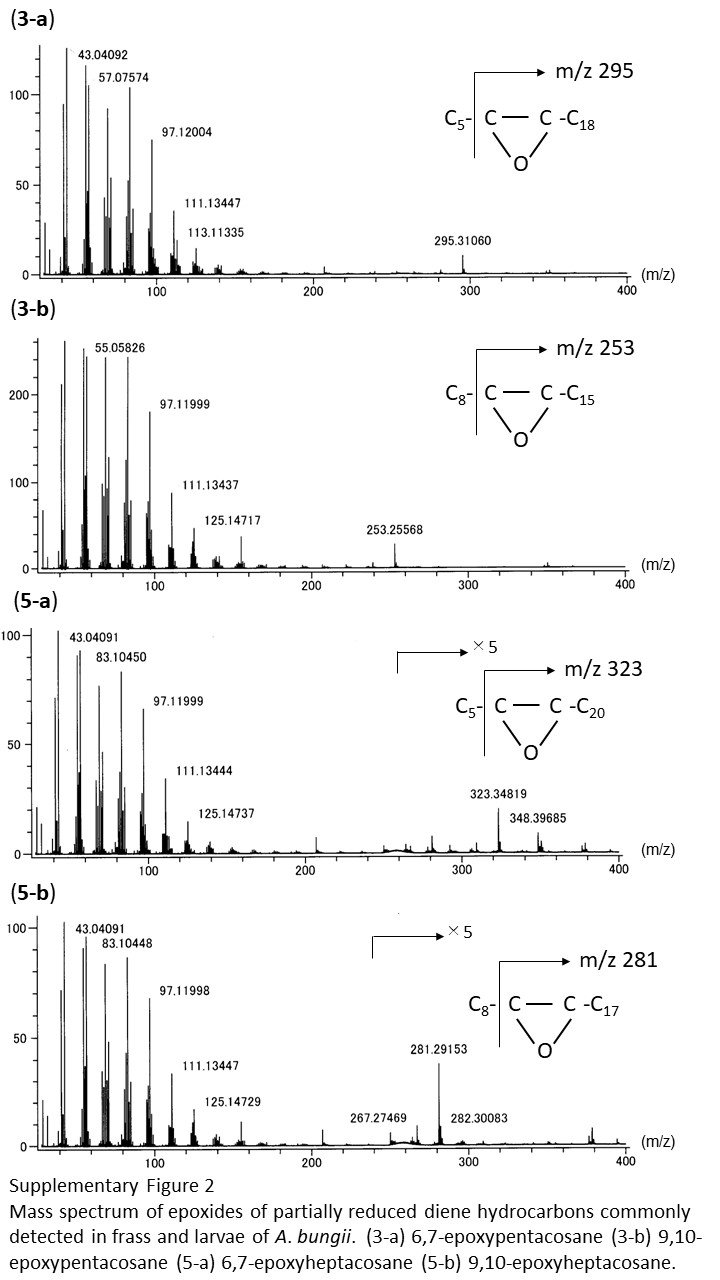

Supplement: Supplementary file 3 — Supplementary Information 3. [file 41598_2023_38835_MOESM3_ESM.jpg]

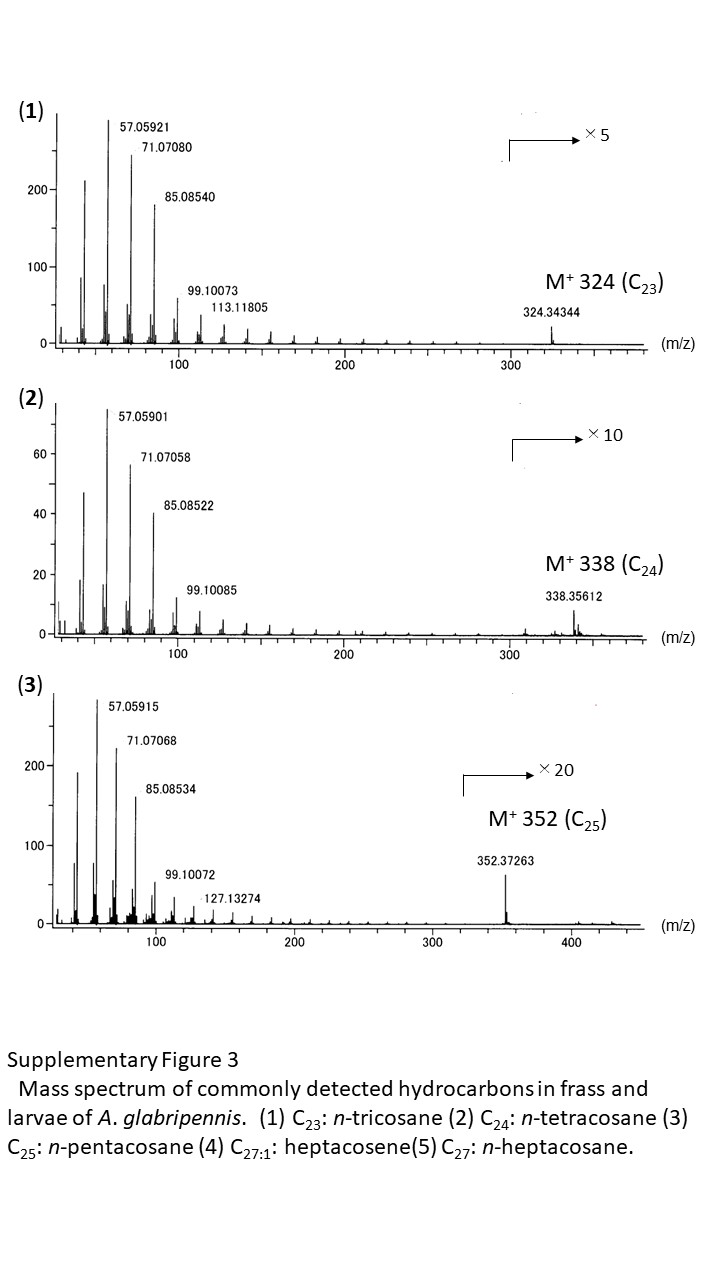

Supplement: Supplementary file 4 — Supplementary Information 4. [file 41598_2023_38835_MOESM4_ESM.jpg]

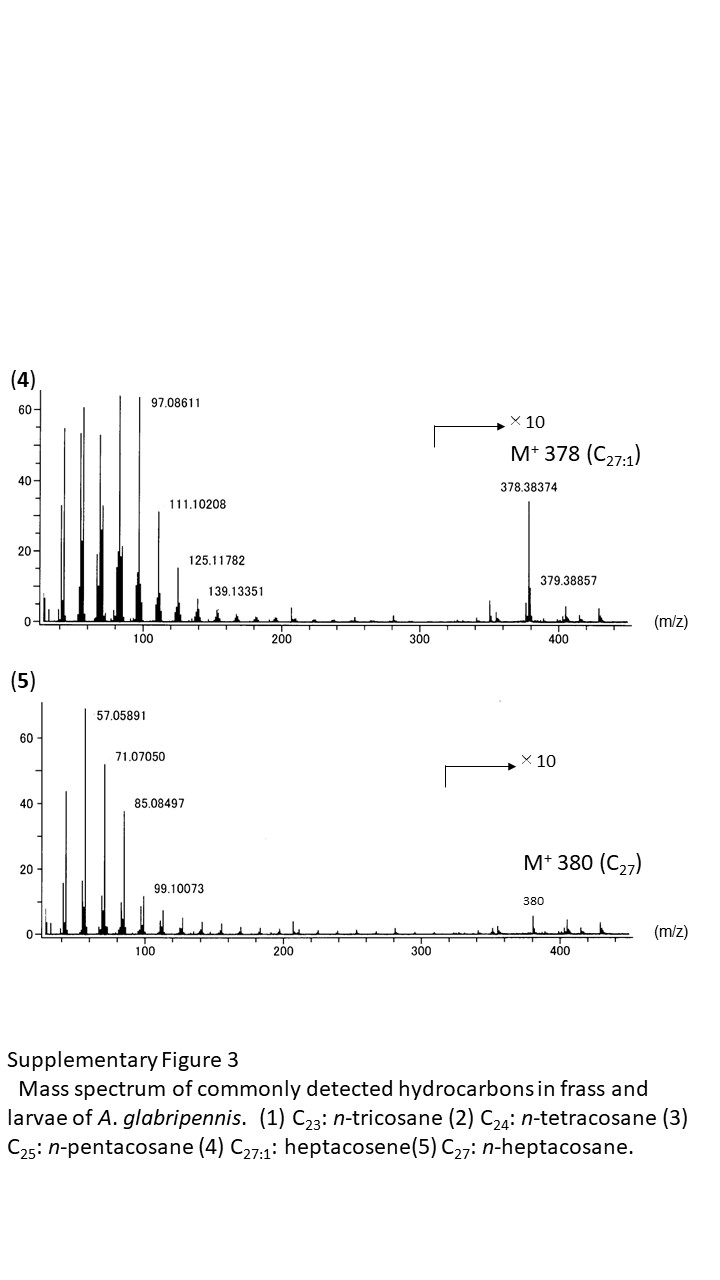

Supplement: Supplementary file 5 — Supplementary Information 5. [file 41598_2023_38835_MOESM5_ESM.jpg]

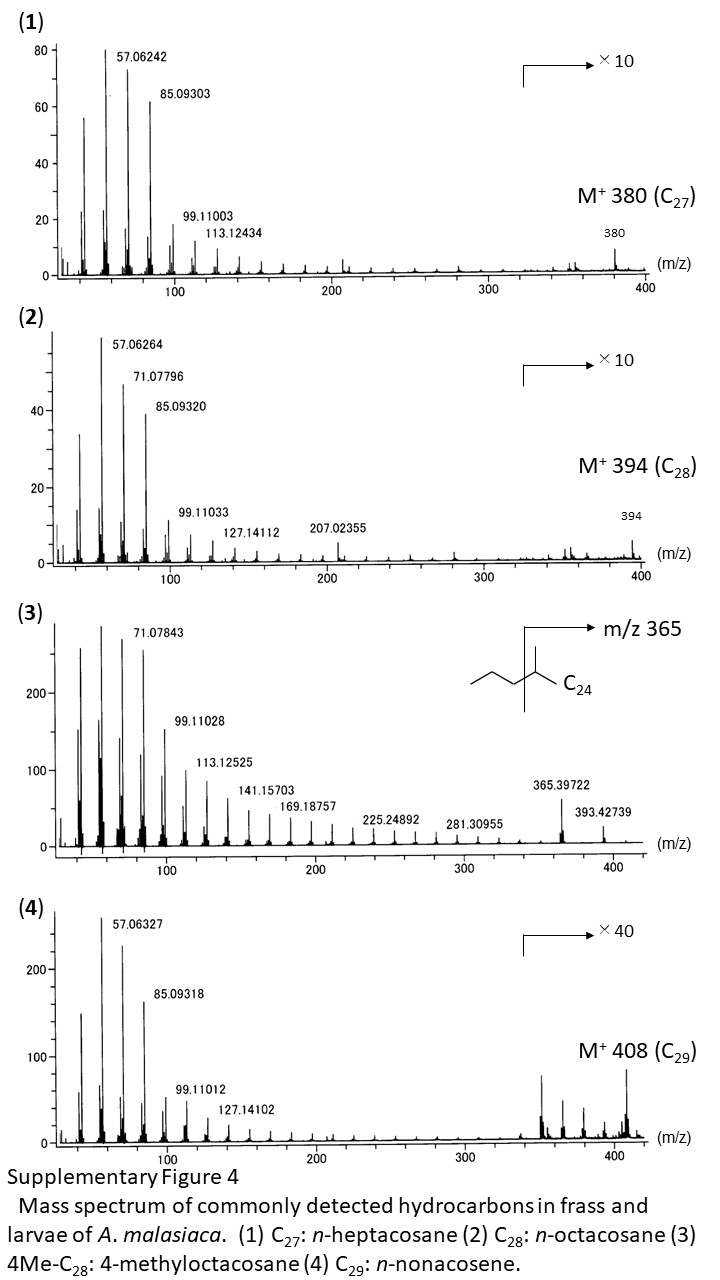

Supplement: Supplementary file 6 — Supplementary Information 6. [file 41598_2023_38835_MOESM6_ESM.jpg]

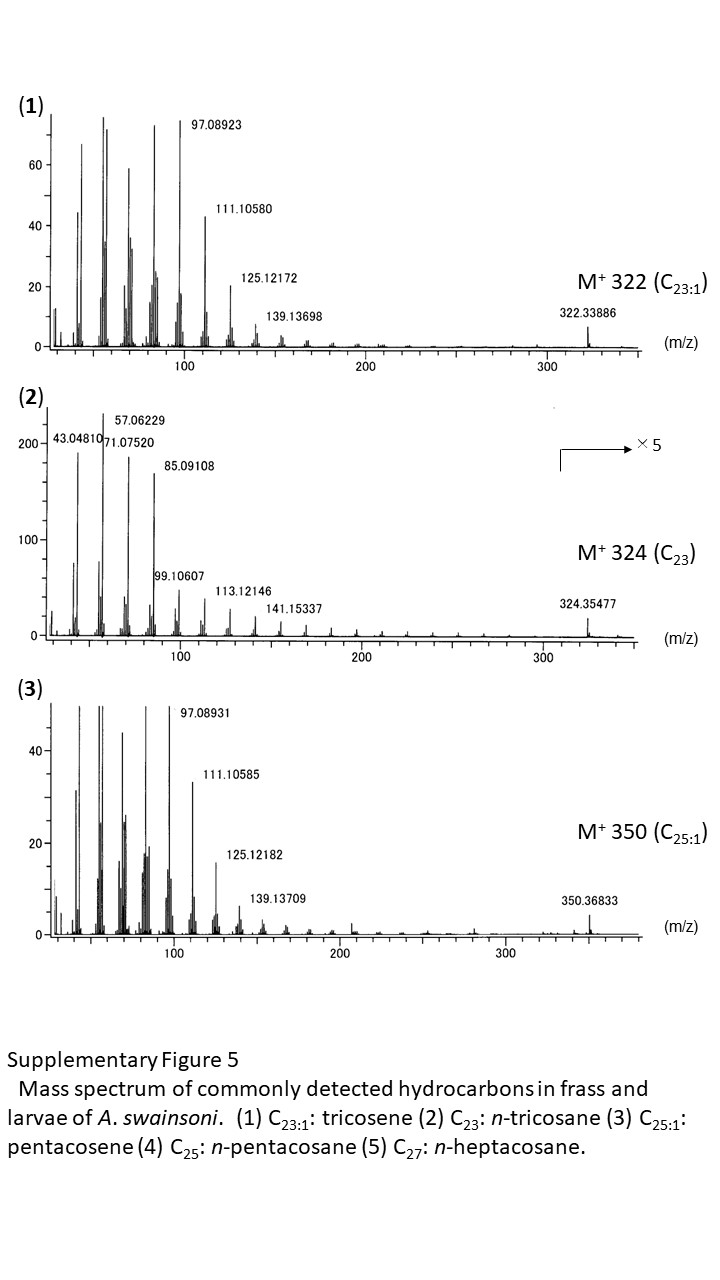

Supplement: Supplementary file 7 — Supplementary Information 7. [file 41598_2023_38835_MOESM7_ESM.jpg]

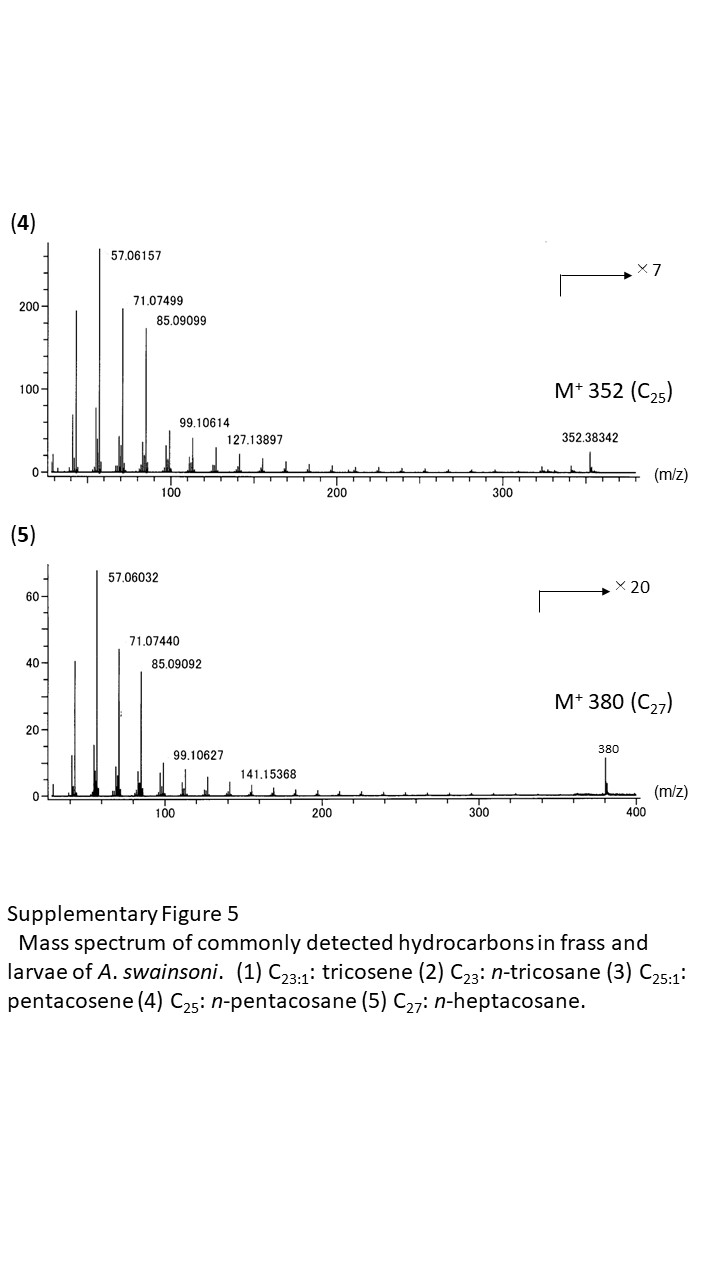

Supplement: Supplementary file 8 — Supplementary Information 8. [file 41598_2023_38835_MOESM8_ESM.jpg]
